# Exploring the Potential of Cleansing Hydrogel and Shampoo with Whey as a Contemporary Approach to Sustainability

**DOI:** 10.3390/gels11050374

**Published:** 2025-05-20

**Authors:** Maja Bjelošević Žiberna, Blaž Grilc, Mirjana Gašperlin, Mirjam Gosenca Matjaž

**Affiliations:** Faculty of Pharmacy, University of Ljubljana, Aškerčeva cesta 7, 1000 Ljubljana, Slovenia; maja.bjelosevic.ziberna@ffa.uni-lj.si (M.B.Ž.); blaz.grilc@ffa.uni-lj.si (B.G.); mirjana.gasperlin@ffa.uni-lj.si (M.G.)

**Keywords:** cosmetology, peptides and proteins, whey, shampoo, cleansing hydrogel, zero waste

## Abstract

Cosmetology is one of the fastest-growing scientific areas, and within it, individual needs and preferences have to be considered. Specifically, cosmetic products with incorporated biological macromolecules, i.e., proteins and peptides, that contribute to improved skin features are gaining in importance. Similar to other fields, cosmetology is also faced with the zero-waste paradigm and strives for a collaboration with other industries. Whey is a co-product in milk production and represents a high environmental burden. In this regard, the idea of the present study was to utilise whey in order to develop sustainable cosmetic products, i.e., cleansing hydrogel and shampoo. The initial phase of the study was dedicated to the development of an optimised hydrogel and shampoo base, followed by whey integration and an in-depth physico-chemical characterisation of both prototypes. In the subsequent phases, particular emphasis was placed on evaluating the potential skin irritancy of the whey-based formulations in vitro, complemented by in vivo assessment on volunteers. The results obtained indicate that the incorporation of whey at concentrations of up to 30% (m/m) is feasible for both formulation types. Moreover, neither product exhibited any irritative effects and a study on volunteers showed that whey has great potential in terms of providing adequate skin hydration. Taken together, all the findings support the development of advanced cosmetic formulations with a zero-waste concept built-in, thus offering a promising platform for cross-sector collaboration, and representing a meaningful step toward potential hydrogel and shampoo commercialisation.

## 1. Introduction

In line with today’s global commitments to sustainability, industries from different sectors are increasingly focused on reducing environmental pollution and achieving zero waste production. The paradigm of the circular economy, which encourages collaboration among different industries, has gained significant importance in recent years [1]. The cosmetics and personal care industry is a science-driven and highly innovative sector with substantial investments in research and development. Concurrently, cosmetology is one of the fastest-growing scientific areas and is increasingly focused on products tailored to individual needs and preferences. As a part of this concept, new or renewed cosmetic ingredients are used to gain improved formulation performance along with enhanced anti-aging, regenerating, moisturising, or cleaning efficacy [2,3]. Proteins, particularly peptides, are one of the most advanced cosmetic ingredients, valued for their high biocompatibility and low risk of allergies. As proteins are large molecules with low water solubility, they are typically used in hydrolysed forms. The modern era of peptide use in skincare began in the early 20th century with yeast-extract peptides being used for wound healing, paving the way for the current active skincare innovations. According to their origin, two groups of peptides and proteins are used in cosmetics: (1) those that structurally and functionally mimic naturally present proteins in the skin and hair (collagen and elastin) and (2) those introduced for their unique skin and haircare benefits [4,5]. These molecules improve skin hydration by forming a protective film on the surface, which reduces transepidermal water loss (TEWL) and enhances skin smoothness and softness.

According to Gorouhi et al. [6], topical bioactive peptides and proteins are categorized in four groups: signal peptides, carrier peptides, neurotransmitter-inhibitor peptides, and enzyme inhibitor peptides. Signal peptides affect the cellular processes characteristic of aging, in general, stimulating the synthesis of skeletal proteins, especially collagen, and, to some extent, also elastin. Carrier peptides facilitate the transfer and stabilisation of trace elements, such as Cu^2+^ and Mg^2+^, vital for healing and enzymatic functions, while neurotransmitter-inhibitor peptides mimic the botulinum toxine mechanism by inhibiting acetylcholine release at the neuromuscular junction. The mechanism of enzyme inhibitor peptides is related to their direct or indirect inhibition of enzymes like superoxide dismutase, hyaluronan synthase 2, and proteinases. Within the group of enzyme inhibitors, several natural peptides are known, such as soja, rice, and silk peptides/proteins [6].

Whey is usually considered as a waste material, with production of 190 million tonnes annually. Whey is a turbid yellowish liquid composed mostly of water, lactose, lipids, minerals, and, above all, with superior protein composition. Whey proteins are a group of soluble milk proteins obtained by precipitating caseins when regulating the pH of milk and comprising β-lactoglobulin (48–58%), α-lactalbumin (13–19%), immunoglobulins (8–12%), serum albumin (5–6%), lactoferrin (2%), lactoperoxidase (0.5%), glycomacropeptide (12–20%), lysozyme, lactoferricin, and cytokines [2]. This protein-rich composition of whey is also beneficial from the cosmetology perspective [7]. For example, whey proteins effectively inhibit skin irritation and help diminish redness, which accelerates skin regeneration after exposure to various stressors, e.g., sun exposure. Additionally, they affect the skin pigmentation and thickness of the epidermis as skin-aging inhibitors [5]. Interestingly, Speer and Amin (2021) explored the potential of O/W emulsion containing whey protein concentrate, chitosan, and different oils on structural responses to temperature, and found out that the addition of whey concentrate influences the formulations capacity to adapt to temperature variations, which is particularly relevant when developing cosmetics tailored to specific climatic or geographical conditions [8]. Whey has also been identified as a potential fermentation medium for producing postbiotics, which can then be integrated into skincare formulations [9]. Among the most valuable whey proteins is lactoferrin, which exhibits antimicrobial/antiviral, anti-inflammatory, and antioxidant activity. Its biological activity is attributed to its iron binding capacity, which, consequently, means it is no longer available for bacteria growth [10,11,12,13]. The amount of whey in cosmetic products varies and ranges from 0.5 to 10%, with the most effective concentrations falling between 0.75 and 3% [10].

As presented above, the existing literature reports some studies focused on the incorporation of whey and, in particular, whey proteins into cosmetic formulations. While such products are also available on the market, the range of formulations containing liquid whey remains extremely limited. Despite our efforts, no cleansing hydrogel incorporating liquid whey was identified, highlighting the innovative aspect of the present research.

The main goals of the present research work were threefold: (i) to contribute to the zero-waste paradigm in the milk industry by exploiting the incorporation of unmodified whey with a full protein composition into cosmetic products (shampoo and cleansing hydrogel); (ii) to evaluate the irritancy potential of both cosmetic products through a pig-ear test and TEWL measurements; and (iii) to estimate the impact of whey-based shampoo and cleansing hydrogel on skin barrier function, i.e., TEWL, SC hydration, erythema and melanin index, and skin gloss in humans. To fulfil these research goals and determine whether whey has potential in terms of the concept of cosmetology and sustainability, the set of presented methods was designed and the results obtained are critically presented hereinafter.

## 2. Results and Discussion

### 2.1. Shampoo Development

Shampoos represent the largest segment of the haircare market and are widely used to clean the hair and scalp by removing soils, such as natural oily exudates and airborne soils, as well as backlogs of hair cosmetic. Typically, shampoos are formulated as an aqueous solution or a dispersion of one or more cleansing additives and other components such as foaming agents, thickeners, conditioners, antidandruff ingredients, colours, fragrances, and pH-adjusters [14]. The initial segment of the research was focused on formulation aspects, with a specific focus on the correlation amongst the surfactant concentrations and physico-chemical properties of the shampoo. Meanwhile, the overall scope was to integrate whey into a previously optimised shampoo composition from two perspectives. First, the consumer-oriented perspective, including the potential impact of whey on the hydration, thickness, and smoothness of the hair, and, second, from a sustainability standpoint, by replacing the water phase with whey, a byproduct of cheese production that is often treated as a waste material.

#### 2.1.1. The Effect of Surfactants’ Concentration

Surfactants are a key component in shampoo and have multifaceted roles, the essential one being hair cleansing, and, notably, contributing to formulation aspects like foaming and viscosity. The surfactants’ mechanisms are diverse, but fundamentally, they are involved in reducing the interfacial tension between water–lipids and water–hair, leading to the formation of spherical droplets of sebum, which are then effectively removed from the hair. Additionally, impurities can be eliminated through the alteration of water-insoluble components within micelles [15]. Surfactants also influence the rheological properties of shampoos, wherein the optimal viscosity facilitates the shampoo dispensing from the container, ensuring its proper application and uniform distribution across the hair. Surfactants dictate the rheological properties of the formulation via the self-association and formation of different three-dimensional structures and mesophases. The mechanism is concentration-dependent; specifically, at lower surfactant concentrations, spherical micelles predominate. As the surfactant concentration increases, elongated rod micelles form, contributing to viscosity. A continued increase in surfactants induces the formation of liquid crystal-based lamellar structures and, ultimately, inverse micelles with a typical viscosity reduction effect are formed. The influence on viscosity is complex and depends on the nature of the surfactants, the addition of co-surfactants (secondary surfactants), and the presence of salts (usually sodium chloride) [15,16,17]. Ensuring the appropriate physico-chemical properties of the shampoo, along with it being non-irritant to the skin, scalp, and eyes, poses a unique challenge.

Within the shampoo development, we initially focused on an appropriate concentration of the primary surfactant (sodium laureth sulfate) and then the proper ratio relating to the secondary surfactants (cocamidopropyl betaine: cocamide diethanolamine), each at fixed concentration of sodium chloride (2.5%, m/m). By varying the sodium laureth sulfate concentration within the range of 5% to 20% (m/m), we observed significant changes in the fluidity of the shampoo. At this stage of the study, fluidity, as a sensory attribute, represented an indirect estimation of product viscosity, namely through the product’s adhesiveness to the container wall or its excessive liquidity. Based on the sensory evaluation, the optimal flow was achieved at a sodium laureth sulfate concentration level of 15% (m/m), where shampoo was smoothly dispensed from our container; moreover, its resistance was observed when placed between two fingers. Our results suggest that at primary surfactant concentrations below 15% (m/m), the flow of shampoos was inappropriately high. The transformation from a spherical to an elongated shape, a factor contributing to viscosity, probably did not occur within this concentration range, despite the addition of salt, which is known to increase micelle size and, consequently, to boost formulation viscosity. On the other hand, the concentration of the primary surfactant, i.e., 15% (m/m), was sufficiently high to form interactions with the complex of secondary surfactants, particularly with cocamidopropyl betaine. This mechanism for increasing viscosity is based on the formation of a gel-like network and bringing elongated micelles closer to each other. A further increase in the concentration of sodium laureth sulfate resulted in a formulation exhibiting a consistency characteristic for semisolids, making the formulation resistant to dispensing with an applied manual force typical for shampoo application. Considering the findings outlined above, for further research, the concentration of sodium laureth sulfate was maintained at 15% (m/m).

Beyond the sustainability paradigm, the intention of this study stems from some reports indicating that the commonly used surfactant cocamide diethanolamine can be associated with the formation of nitrosamines, recognised as human carcinogens. Consequently, its incorporation in cosmetic products has to be considered with special care. Given its significant contribution to the properties of shampoo, finding a suitable replacement poses a considerable challenge. Cocamide diethanolamine is a non-ionic surfactant with a strong thickener effect in combination with sodium chloride and characterised as a viscosity agent [18]. In the context of developing a shampoo with a minimal amount of the controversial cocamide diethanolamine, we also tested its combinations with cocamidopropyl betaine in various ratios (Table 1).

Cocamidopropyl betaine is an amphoteric surfactant, which, upon lowering the pH of the formulation, acquires its cationic nature and interacts with the negatively charged sodium laureth sulfate [19]. In our experiment, shampoo with cocamide diethanolamine and cocamidopropyl betaine at 2.5:0.5 mass served as the reference for comparing the viscosity of subsequent formulations with reduced cocamide diethanolamine concentration. The obtained results revealed that shampoos with equal concentrations of both surfactants (1.5:1.5 mass), as well as the one with a higher concentration of cocamidopropyl betaine (0.5:2.5 mass), exhibited comparable flow behaviour when dispensing from the container as the reference. This suggests that despite reducing the concentration of the non-ionic surfactant, there was no critical shortening of wormlike micelles. Simultaneously, we hypothesised that the surface charge of micelles did not exceed a range that would induce the intense electrostatic repulsive interactions responsible for a drop in viscosity. An opposing trend was observed in a formulation without cocamide diethanolamine, therefore, a complete replacement of cocamide diethanolamine with cocamidopropyl betaine is not suitable for tested formulations. At this point, it is worth reiterating that cocamide diethanolamine is a multifunctional ingredient, acting as a viscosity enhancer, foaming agent, and foam stabiliser. As such, replacing it with a single alternative is often challenging and, typically, a comprehensive reformulation strategy is needed. The use of cocamide diethanolamine in cosmetic formulations is subject to regulatory limitations, and its inclusion depends on the characteristics of other ingredients as well as the specific type of cosmetic product. Thus, a shampoo with a cocamide diethanolamine to cocamidopropyl betaine ratio of 0.5:2.5 was selected as a leading formulation for whey incorporation.

#### 2.1.2. The Effect of Whey Concentration

**Figure 1:**
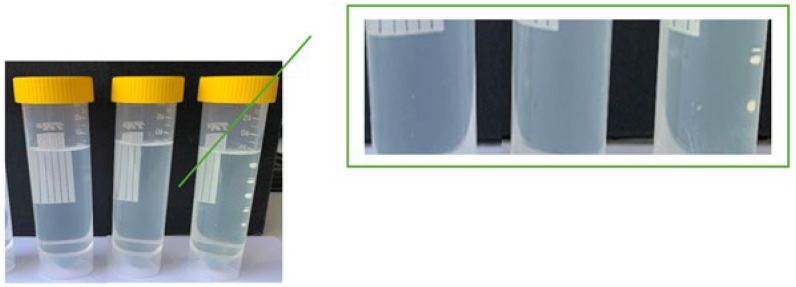
Shampoos with different whey concentration incorporated (from left to right: 10, 20, and 30%, m/m).

As whey exhibits a broad spectrum of positive effects on skin features, our concept additionally embraces sustainability and environmental considerations, which are accomplished through the substitution of water with whey. Due to the intensive energy-consuming process of obtaining dried whey, something that contradicts the eco-friendly purpose of the article, only liquid whey was studied. We incorporated whey into the shampoo at concentrations of 10, 20, and 30% (m/m), which was achieved by reducing the amount of purified water. As shown in Figure 1, the addition of whey led to a yellow discoloration of the shampoos, which became increasingly intense with higher whey concentrations. The same figure also demonstrates that shampoos maintained their viscosity without visible sedimentation or protein aggregation, regardless of whey concentration. We presume the absence of interactions between whey components and sodium chloride or surfactants, leading to a preserved three-dimensional structure of whey proteins. Also, the thickener ability of secondary surfactants was preserved, most likely due to maintaining a stable pH despite the addition of whey. To maximise yield and to fully utilize whey’s cosmetic benefits, the shampoo with the highest whey concentration, i.e., 30% (m/m), was chosen as the final formulation for stability, in vitro, and in vivo performance evaluation.

### 2.2. Cleansing Hydrogel Development

Hydrogels comprise a polymer network (i.e., solid phase) that swells and retains large amounts of water (i.e., liquid phase) by maintaining three-dimensional structures [20]. Cleansing hydrogel with whey incorporated would represent an appropriate type of cosmetic product for oily and acne-prone skin due to its lactoferrin antibacterial action. Additionally, substituting the water phase of hydrogels with whey represents a potential strategy for reducing water consumption. Before the incorporation of whey, the optimal composition of hydrogel needed to be defined.

#### 2.2.1. The Effect of Xanthan Gum and Decyl Glucoside Concentration

Xanthan gum is a biodegradable and biocompatible polysaccharide often used as a macromolecule forming a gel structure [21]. From a rheological perspective, hydrogels can be characterised as non-Newtonian systems, where viscosity reflects the mechanical resistance of the gel network to the imposed shear rate. Our results on varying concentrations of xanthan gum (Table 2) revealed that a hydrogel with 0.5% and 1% (m/m) exhibited excessive flow, whereas a concentration of 1.5% (m/m) required a higher force to initiate the flow of the hydrogel, demonstrating an appropriately viscous consistency suitable for application to the skin. It was presumed that viscosity building occurred due to the higher proportion of solid content in the hydrogel with the 1.5% (m/m) xanthan gum concentration. As we aimed to develop a cleansing hydrogel, the incorporation of a surfactant was essential. As an excipient with cleansing function, a non-ionic decyl glucoside was selected and tested in three different concentrations (10, 15, and 20%, m/m). Decyl glucoside is the one most commonly used among the alkyl glucosides, is often present in rinse-off products at concentrations ranging from 0.3 to 30%, and has good dermatological compatibility [22]. However, during the development of our hydrogel, a drawback related to decyl glucoside was observed. Specifically, concentrations of 15% and 20% (m/m) had a negative impact on gel thickening, resulting in decreased viscosity, along with turbidity occurrence due to the pale-yellow coloration of the decyl glucoside and the intense foaming of the hydrogel. Another challenge was the elevated pH (~10.5) resulting from the addition of decyl glucoside, irrespective of its concentration. While manageable, this required the addition of a higher amount of citric acid to achieve the target pH (5.2). To strike a balance between the cleansing effectiveness and physico-chemical properties of the hydrogel, the concentration of decyl glucoside was set to 10% (m/m).

#### 2.2.2. The Effect of Whey Concentration

**Figure 2:**
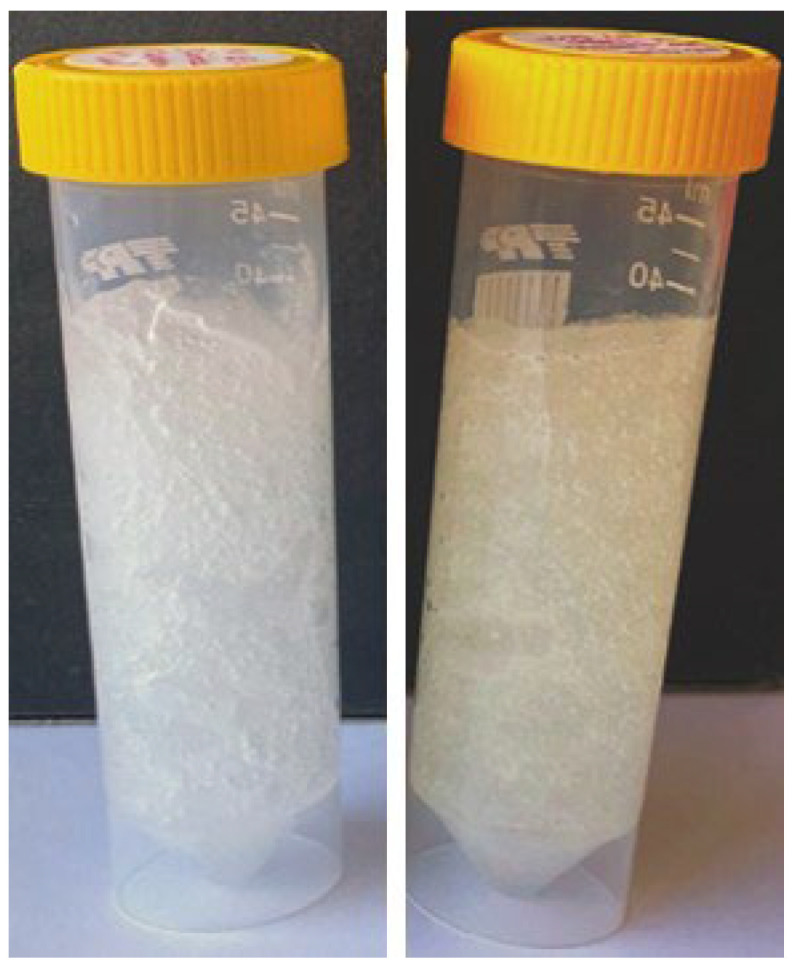
Hydrogel with no whey (**left**) and 30% (m/m) of whey incorporated (**right**).

As stated, the incorporation of whey into the cleansing hydrogel was linked to its antibacterial effects, attributed to the presence of lactoferrin, and aimed to contribute to environmental care by preventing the disposal of significant amounts of whey. Replacing the water phase with whey from 10% to 30% (m/m) did not result in any differences between the samples, indicating that the incorporation of the highest whey concentration is feasible. A comparison between samples with and without whey incorporated revealed that the acidic nature of whey slightly lowered the pH of the hydrogel, namely from 6.4 to 6.0 (before pH adjusting to 5.2), and induced a yellow coloration (Figure 2).

It is worth emphasising here that, in the continuation of the study, the viscosity and pH of the selected products were assessed in vitro using a rheometer and pH meter.

### 2.3. Stability Evaluation

The stability of cosmetic products is a critical parameter, since it ensures their safety profile during the set shelf-life, and, to some extent, correlates to cosmetic active ingredients’ stability and, consequently, efficacy. The purpose of stability studies is to ensure the physical, chemical, and microbiological quality of cosmetic products in accordance with the prescribed quality standards, as well as their functionality and aesthetic appearance under prescribed storage conditions. In the stability study, the most optimal formulations were included, i.e., a shampoo composed of 15% (m/m) sodium laureth sulfate, cocamide diethanolamine and cocamidopropyl betaine in a ratio of 0.5:2.5, and 30% (m/m) whey (Table 1, F10) and a hydrogel with 1.5% (m/m) xanthan gum, 10% (m/m) decyl glucoside, and 30% (m/m) whey (Table 2, F8). To assess the potential impact of whey, the corresponding whey-free shampoo and hydrogel formulations were also subjected to stability evaluation. All formulations underwent organoleptic and physico-chemical (pH, viscosity) evaluation before stability testing and at predetermined time points during the six-week stability study period. Such an approach enables the comparison of product characteristics and allows for potential modifications during this time.

**Figure 3:**
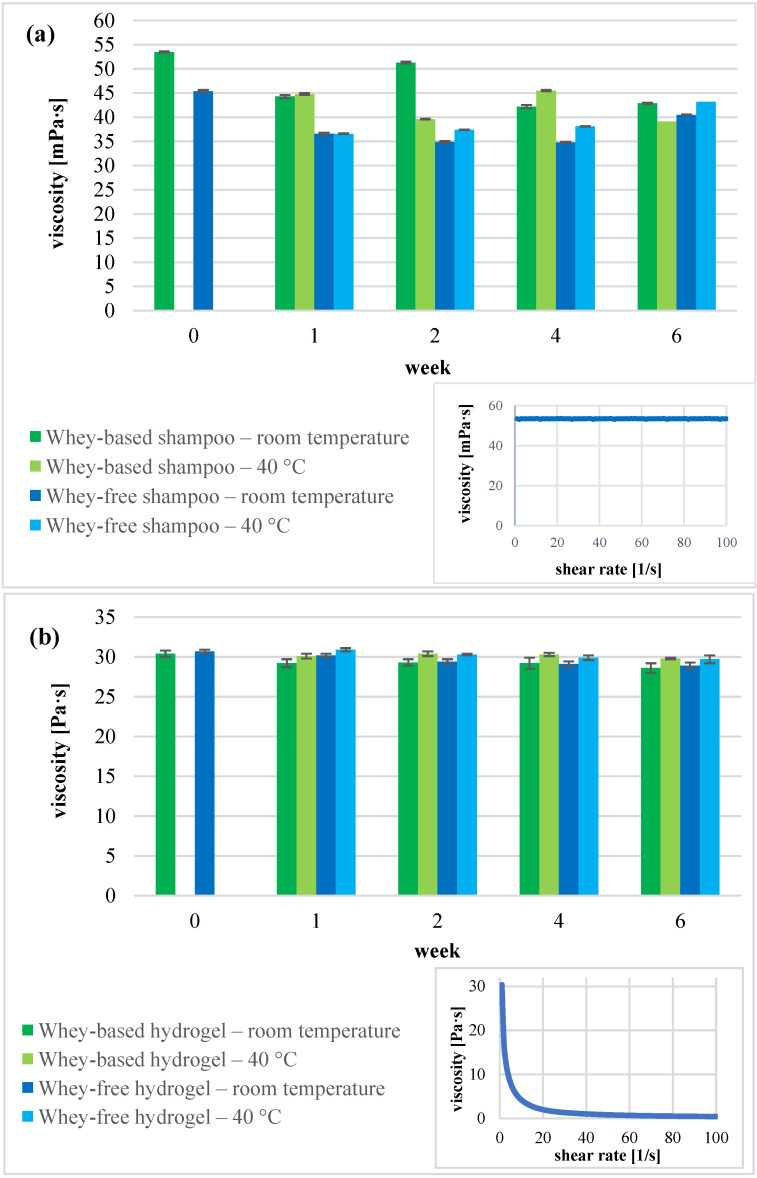
Rotational viscosity of whey-based and whey-free samples at shear rate 1 s^−1^*,* i.e., of (**a**) shampoo and (**b**) cleansing hydrogel before stability study and within 6 weeks at 25 °C and 40 °C. Inset: representative viscosity flow curve of (**a**) shampoo and (**b**) cleansing hydrogel.

When comparing the organoleptic properties of shampoo with and without whey incorporated, both immediately after preparation and during the stability study, the most notable issue was the impact of whey on colour and odour. Namely, shampoos with whey were slightly yellowish, and had whey odour. None of the samples expressed the visible signs of instability. Based on rheological behaviour, the Newtonian behaviour was considered for the shampoos (Figure 3a inset) since the viscosity did not alter considerably during different share rates, indicating shampoos as being colloidal solutions. Detailed assessment of viscosity measurements results, which are graphically presented in Figure 3a, revealed that whey-based shampoo exhibited a marginally higher viscosity at the starting point compared to a whey-free counterpart, namely, 53.5 mPa·s and 45.4 mPa·s, respectively. This trend remained consistent throughout the stability study. Although minor viscosity fluctuations were observed over time, a statistically significant difference (*p* = 0.02) emerged exclusively at the week 2 time-point under room temperature storage conditions, with the whey-based formulation demonstrating a markedly higher viscosity (51.3 mPa·s vs. 34.9 mPa·s, respectively). The pH level is also a critical quality attribute in terms of stability. The statistical analysis revealed significant differences in pH for shampoo with whey incorporated and stored at room temperature (*p* = 0.003) and at 40 °C (*p* = 0.002), respectively, when compared to whey-free shampoos. It could be speculated that some chemical degradation mechanisms in terms of whey took place, but since the pH difference did not exceed the limit of 0.5, specifically, starting from an initial value of 4.7, the pH fluctuated within a narrow range between 4.5 and 4.9, we concluded that prepared shampoos with whey were stable from an organoleptic as well as a physico-chemical point of view.

All prepared hydrogels performed as weak gels, showing shear thinning behaviour (Figure 3b inset). As shown in Figure 3b, hydrogels as semisolid formulations exhibited a higher viscosity range compared to shampoos. The incorporation of whey has no impact on viscosity, i.e., 30.7 Pa·s was obtained for a whey-free hydrogel, and 30.4 Pa·s in the case of whey. Values remained almost constant during the stability study timeframe irrespective of storage conditions, being within 28.9 and 30.9 Pa·s. Regarding the pH value, no significant differences were observed during the stability study, namely, it varied within the range of 5.1 to 5.4, starting from an initial value of 5.2., indicating the high stability of hydrogel formulations, with only slight colour changes to yellowish.

### 2.4. In Vitro and In Vivo Evaluation

#### 2.4.1. In Vitro Skin Irritation Evaluation

The forefront of cosmetic product development must always prioritise their safety, with a focus on skin irritation. In light of this, prior to conducting an in vivo study on volunteers, the irritative potential of shampoo and hydrogel with and without whey incorporated was assessed through in vitro pig-ear test. The criterion for defining a product as potentially irritating to the skin was an absolute increase in TEWL ≥ 6 g/hm^2^ in comparison to the baseline value.

Initially, we tested shampoo formulations, each in three parallel systems. Specifically, the irritation profile of each of the two formulations—with and without whey—was assessed across three separate Franz diffusion cell systems. The baseline values of all six skin samples were comparable, ranging from 13.49 to 19.38 g/hm^2^ and indicating a normal healthy state of the skin barrier. In the case of the shampoo with whey incorporated, 10 min after the removal of the formulation, TEWL values increased by a maximum of 5 g/hm^2^ (i.e., to 21.49 and 21.56 g/hm^2^) and stabilized within the baseline range after 20 min for two parallel systems, while a significant increase in TEWL (to 30.58 g/hm^2^) was observed for one parallel system, with the final measurement being, likewise, comparable to the baseline (12.42 vs. 13.49 g/hm^2^, respectively). The mentioned trend was also observed in the evaluation of the shampoo without whey incorporated, where, 10 min after withdrawing the formulation, all three parallel systems showed significantly higher TEWL values, which stabilized near to baseline values, i.e., 12.44 and 21.49 g/hm^2^, at the final measurement point. Despite careful removal of the tested formulation, increased TEWL values after 10 min could be attributed to the formulation residual on the skin, resulting in falsely elevated TEWL values. According to statistically non-significant changes after 20 min of formulation removal (*p* > 0.05), it was, therefore, concluded that shampoo with and without whey incorporated did not induce skin irritation.

Following the same procedure, the impact of hydrogels on TEWL and, consequently, their skin irritation profile, was assessed. The average baseline value of TEWL was 21.97 g/hm^2^, indicating a slightly compromised skin barrier function. However, as no visible damage was observed on the skin, and relative changes in TEWL were focused, we conducted a test. In the case of the hydrogel with whey incorporated, an increase in TEWL (by 4.51 g/hm^2^) occurred only in one parallel system after 10 min following the removal of the formulation, while all other TEWL values were similar to or even slightly below baseline values. A similar trend was observed for the hydrogel without whey incorporated, where, despite an average increase in TEWL for 5.02 g/hm^2^ in the first measurement after the removal of the formulation, the final TEWL values reached the range of baseline values. Considering that the increase in TEWL did not exceed 6 g/hm^2^, and there were no statistically significant differences among the results, the irritative behaviour of both hydrogels was assessed as negligible.

#### 2.4.2. In Vivo Evaluation of Efficacy of Shampoo and Cleansing Hydrogel

The barrier function of the skin is inseparably associated with the SC features and, as such, determined by the integrity of the SC itself. Corneocytes, along with the lipid matrix, represent a crucial component of the skin barrier because they influence the absorption of cosmetic ingredients into the skin. A functional barrier is a prerequisite for healthy skin and can be assessed through bioengineering measurements including TEWL, SC hydration, erythema and melanin index, and skin gloss [23,24,25]. Although we recognise that assessing the effectiveness of shampoos when applied directly to the scalp would provide added value to the study, we aimed to evaluate the effects indirectly by measuring skin hydration and TEWL on the forearm. This approach allowed us to assess whether the set surfactants’ combination in the final formulations could contribute to skin redness and potential skin over-drying. Ultimately, we aimed to compare the effects of the shampoo on SC hydration and TEWL with those of the hydrogel. In the context of hydrogels, their cleansing performance was also examined through the measurements of skin gloss.

The skin barrier function can be disrupted by both chemical and mechanical factors, resulting in increased TEWL values. Surfactants, widely prevalent in cosmetic products, are often presumed to be chemical irritants to the skin. However, the effectiveness of both shampoo and cleansing hydrogel is strongly dependent on the action of surfactants. With TEWL being a sensitive indicator of skin barrier integrity, TEWL measurements were initially performed to detect any potential negative impact of the formulated products. The results of the TEWL assessments obtained within the entire study course are outlined in Table 3 as mean baseline values and mean values after exposure to tested formulations. Generation of a 95% confidence interval with a probability level of 0.05 revealed comparable baseline TEWL levels among the study participants, thus, the impact of the shampoo and hydrogel was evaluated considering the average change in post-basal TEWL compared to the basal value. Since neither shampoo nor hydrogel cause significant alternations in TEWL values, we assumed that the composition of both tested products was suitable as it did not compromise the healthy state of the skin barrier. It is valuable to mention that the increase in TEWL was consistently higher in the case of the product without whey incorporated, indicating that whey beneficially regulates the skin barrier function as a response against chemical factors, and supports its protective role, although the TEWL differences between products with and without whey incorporated were, however, insignificant.

Dry skin is a prevalent and burdensome condition that may reflect as transient episodes of dryness and itchiness or could be related to severe skin diseases [26]. Augustin et al. [26] have shown that the prevalence of dry skin increases with age, reaching almost 30% in working adults and averaging around 60% in the elderly [26]. In line with this, younger women of a comparable age (20 to 25 years old) were included in our study. The corneometer, which was used to monitor the SC hydration level, operates on the principle of electrical capacitance measurements, leveraging the phenomenon that water exhibits a significantly higher dielectric constant compared to most other substances. As a result, readings above 45 arbitrary units (a.u.) indicate normally hydrated SC, while values below 30 a.u. reflect a low level of SC hydration. Baseline measurements indicated that the majority of volunteers (85%) had “very dry” skin, in the 12.77 to 29.63 a.u. range. The rest of the values were just slightly above the threshold of 30 a.u. (32.67 a.u.), indicating “dry” skin conditions. A more detailed evaluation of the results in Table 4 reveals a significant increase in forearm hydration following exposure to both tested products irrespective to the inclusion of whey. Specifically, the average differences between the baseline capacitance and the post-basal values for shampoo with and without whey incorporated were 3.83 a.u. (*p* = 0.0005) and 3.27 a.u. (*p* = 0.003), respectively. Moreover, the differences in hydration level were even higher after exposure to hydrogels, namely 21.61 a.u. (*p* = 9.74 × 10^−7^) in the case of hydrogel with whey incorporated, and 20.94 a.u. (*p* = 5.24 × 10^−7^) after exposure to the whey-free hydrogel. We presume that the more pronounced hydration effect of the hydrogel could be attributed to the higher concentration of glycerol as opposed to the amount of propylene glycol in the shampoo, with both acting as humectants with high SC hydration-retention capacity. The mechanism of humectants is accomplished by water absorption as well as interactions with the SC lipid structures or proteins, altering their water-binding capacity [27]. It should be stated here that we took special care to conduct measurements on skin completely free from any product residues. The study also aimed to test the contribution of whey in terms of SC hydration improvement. The literature data state that whey proteins mimic the component of a natural moisturising factor, thus contributing to well-hydrated skin [28]. Our results, as presented in Table 4, suggest no statistical differences between shampoo/hydrogel with and without whey incorporated in terms of enhancing the hydration level. It is necessary to emphasise the fact that both shampoo and hydrogel with whey contained significantly less free water in their composition, as it was replaced with whey. Therefore, SC hydration increased to a comparable extent, as in products with a full water content. We can say that whey has great potential in terms of providing adequate skin hydration.

**Figure 4:**
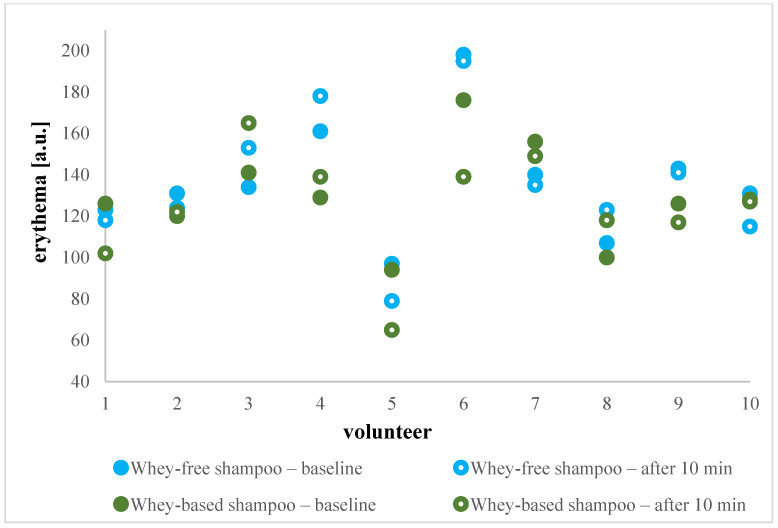
Absolute erythema values in a.u. before and after whey-based and whey-free shampoo application for each of 10 volunteers individually.

**Figure 5:**
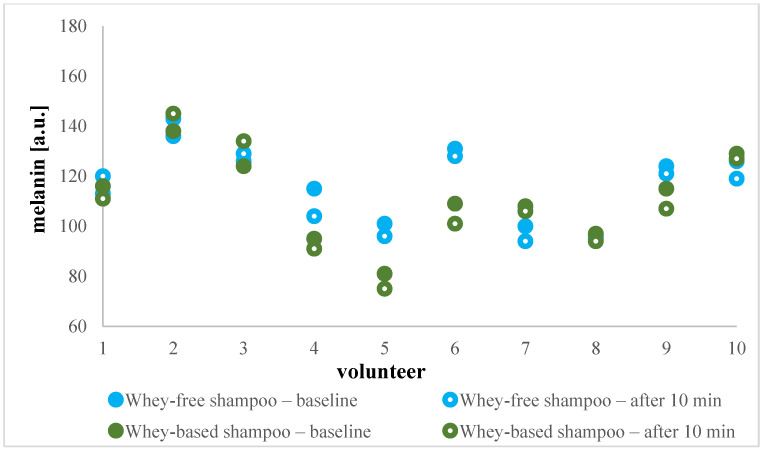
Absolute melanin values in a.u. before and after whey-based and whey-free shampoo application for each of 10 volunteers individually.

Considering this, the in vivo study was continued by measuring the erythema index and melanin index. Erythema can be a consequence of various factors, including external triggers, immune reactions, or is an indicative of an inflammatory skin response [29]. The term erythema refers to the redness of the skin, which occurs due to the dilation of capillaries in response to skin irritation or injury [30]. As it incorporates factors for assessing skin pigmentation and colour, simultaneously with erythema index, the melanin index is usually measured. The mexameter measuring system employed in our study emits three specific wavelengths of light, enabling the quantification of erythema and melanin levels by assessing the light reflection from the skin. Given variations in baseline values, namely from 94 up to 198 a.u. for erythema, and in a range 93–136 a.u. for melanin level, both indices were considered in a person-specific way. As depicted in Figure 4 and Figure 5, erythema and melanin values were preserved or even decreased between basal and post-basal values for all of the shampoos, allowing us to speculate on the regenerative impact of whey. While this observation would certainly warrant further investigation, it can be stated that all tested formulations were associated with good skin tolerability. This was further supported by the fact that none of the volunteers reported any adverse effects on the treated skin area. Thus, it was reaffirmed that novel shampoo composition, including added whey, poses low irritation potential.

**Figure 6:**
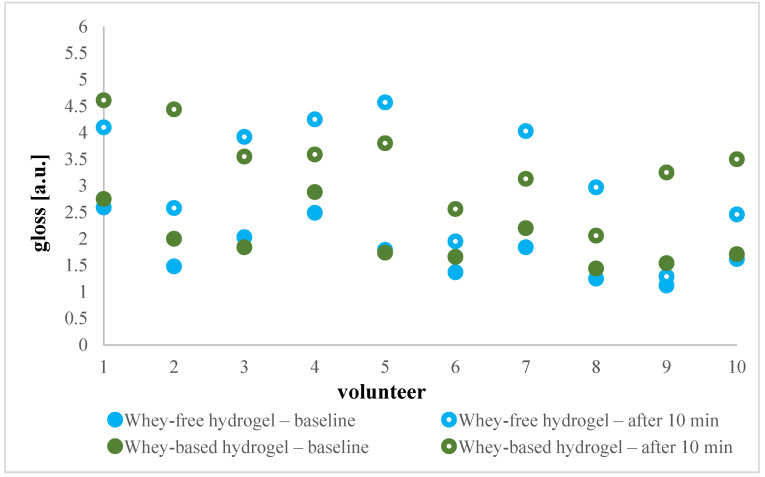
Absolute gloss values in a.u. before and after whey-based and whey-free cleansing hydrogel application for each of 10 volunteers individually.

Skin gloss can be quantitatively characterised based on analysing the reflection of light, namely, the measurement system built into the mexameter enables quantification of direct as well as diffuse reflected light. Directly reflected light reveals the gloss of the skin, while diffusely reflected light represents the portion of light concerning the individual’s surface-specific skin properties [31]. Our measurements (Figure 6) showed a significant difference between baseline and post-baseline values with an increase in skin gloss after the application of hydrogel. In detail, the baseline values of skin varied between 1.12 and 2.88 a.u., and the values after exposure to hydrogel were up to 4.61 a.u. No substantial changes were observed between the hydrogel with and without whey, except for individuals anonymised as numbers two and nine. While the perception of skin gloss is subjective, it is generally accepted that completely glossless skin appears dull and reflects inadequate care.

## 3. Conclusions

The present study demonstrates the feasibility of utilising liquid whey as a sustainable alternative to water, leading to the successful development of a whey-based shampoo and cleansing hydrogel. This innovative substitution not only enhances product functionality but also reflects a commitment to environmental sustainability and the zero-waste paradigm. However, upgrades, including the switch of anatomical testing site, wider group of volunteers and study extensions, together with implementation of modern analytical techniques, microbiological testing, and packaging design, have to be prioritised in the follow-up study. Addressing these considerations within the framework of regulatory compliance will be crucial for advancing the commercial potential of both whey-based cosmetic products.

## 4. Materials and Methods

### 4.1. Materials

The whey was obtained from domestic milk industry in a liquid form, stored at 4 °C and used within three days. Shampoo ingredients: sodium laureth sulfate, sodium chloride, and citric acid (Merck, Darmstadt, Germany), cocamidopropyl betaine (Evonik industries, Essen, Germany), cocamide diethanolamine (Making Cosmetics Inc., Redmond, Washington, DC, USA), and propylene glycol (Fluka chemie GmbH, Buchs, Switzerland). Hydrogel ingredients: glycerol (Farmalabor, Canosa di Puglia, Italy), xanthan gum (Merck KGaA, Darmstadt, Germany), decyl glucoside (Magnolija, Ljubljana, Slovenia), and almond oil (A.C.E.F., Fiorenzuola d’Arda, Italy). The purified water was from University of Ljubljana, Faculty of Pharmacy, Ljubljana, Slovenia.

### 4.2. Methods

#### 4.2.1. Cosmetic Product Development

##### Optimisation of Shampoo Formulation

Different shampoo formulations with composition presented in Table 1 were prepared. First, sodium laureth sulfate was weighed in a beaker and heated to 60 °C in water bath. In the second part, water solution of surfactants, propylene glycol, and sodium chloride were heated to the same temperature. Both solutions were mixed together and cooled down during stirring. Then, liquid whey was added. Finally, the pH was adjusted to 4.7 with citric acid. The sample with no whey incorporated was prepared as a control (F6 in Table 1).

##### Optimisation of Cleansing Hydrogel Formulation

For preparation of cleansing hydrogel formulations (Table 2), glycerol (sugar alcohol) as humectant and anionic polysaccharide xanthan gum, which serves as gel forming substance and stabiliser, were firstly mixed. Mixing was carried out for up to 10 min at controlled room temperature (24.0 ± 1.0 °C), and was dependent on mixing mass, i.e., the amount of xanthan gum (0.5, 1, 1.5%, m/m) at constant glycerol rate (7.5%, m/m), considered that 100 g of hydrogel was prepared in each case. Then, purified water (dispersion medium) was added. Separately, non-ionic surfactant decyl glucoside and almond oil (emollient) were slowly mixed, followed by addition of the first part. Then, the whey was incorporated for the respective samples.

##### Viscosity Measurements

Viscosity measurements were performed by a rotational method using modular rheometer Physica MCR 301 (Anton Paar, Graz, Austria) with coaxial cylinder (CC27/T200/SS) for shampoos, while a conical disk diameter of 49.961 mm and a cone angle of about 2.001° was used for hydrogels. The shear rate was increased from 1 to 100 (1/s) with measurements carried out at 25 °C and results evaluated using Physica software Rheoplus/32V3.62.

##### pH Measurements

For pH determination, Mettler Toledo (Columbus, OH, USA) Seven Compact pH meter with plastic electrode calibrated against three standards, at pH 4.01, 7.01, and 9.00, was used. All measurements were performed in a temperature range between 23 and 25 °C. As a result, average value of three measurements was given.

##### Organoleptic Evaluation

Among organoleptic parameters, appearance, colour, odour, and phase separation were evaluated and described as modified/unchanged.

##### Stability Study

The stability study was performed as short-term accelerated test. Shampoos and cleansing hydrogels were exposed to room and elevated (40 °C) temperatures for six weeks. Organoleptic as well as physico-chemical properties of shampoos and hydrogels were evaluated before stability study and after 1, 2, 4, and 6 weeks [32].

#### 4.2.2. Evaluation of Irritancy Potential of Shampoo and Cleansing Hydrogel

The irritancy potential of selected shampoos and hydrogels was estimated based on TEWL measurements. The TEWL was determined using the nonperfused pig-ear test, following the European Centre for the Validation of Alternative Methods-recommended procedure [33].

Pig ears were obtained from a local abattoir and washed under tap water. The skin samples were prepared by carefully removing the whole skin from the underlying cartilage. All skin samples were inspected for visible skin lesions, with only intact skin used for the test. Individual sets of measurements were performed using skin samples from the same ear or at least from ears of the same animal in order to minimise inter-individual differences on variability of results. The skin (2 cm × 2 cm) was mounted on Franz diffusion cells that contained 8 mL 0.9% aqueous sodium chloride solution in receptor chamber. The Franz cells (n = 3) were put into a water bath at 37 °C and allowed to temperate for 1 h, establishing a temperature gradient with ambient room temperature resulting in skin surface temperature of ~32 °C. The basal TEWL was then measured (MPA 5 Tewameter; Courage + Khazaka electronic GmbH). All TEWL measurements were performed in a controlled environment, i.e., at room temperature of 24.0 ± 1.0 °C, and relative humidity of ~45%. Subsequently, approximately 200 mg of the tested formulation was put onto the skin surface. After 10 min, the tested formulation was removed, and the skin surface was dried with a cotton swab. The TEWL was then measured after 10 and 20 min following formulation removal, and the absolute increase in TEWL was calculated as the difference between basal TEWL and that after the formulation exposure. The criterion for classifying the formulation as potentially skin irritating was an absolute increase in TEWL ≥ 6 g/hm^2^ [34]. Statistical analysis of results was carried out using the independent samples Student’s *t*-test. Significance was tested at the 0.05 level of probability.

#### 4.2.3. In Vivo Study: Efficacy Evaluation of Shampoo and Cleansing Hydrogel

##### Initial Segment

The protocol for the in vivo study and other essential documents were approved by the National Medical Ethics Committee of the Republic of Slovenia (approval number: 0120-232/2023/6). The study was performed according to the general principles of the Declaration of Helsinki with all its amendments. The study was conducted on 10 healthy females in the age range of 20 to 25 years, at the University of Ljubljana, Faculty of Pharmacy, as a double-blind, interventional study. All volunteers who participated in the study were informed about the purpose and course of the research verbally in an understandable way. All participants confirmed that they participated voluntarily, understood the information given and signed a written informed consent. All personal information of the participants was treated as highly confidential. Also, all participants were informed that they could withdraw from the study at any time without giving a reason.

One week prior to the study and throughout the entire study period, volunteers were instructed not to use cosmetic products, except shower gel. Subjects were requested not to engage in intensive physical activity, consume any caffeinated beverages or smoke within two hours prior to instrumental measurements.

##### Study Protocol

**Figure 7:**
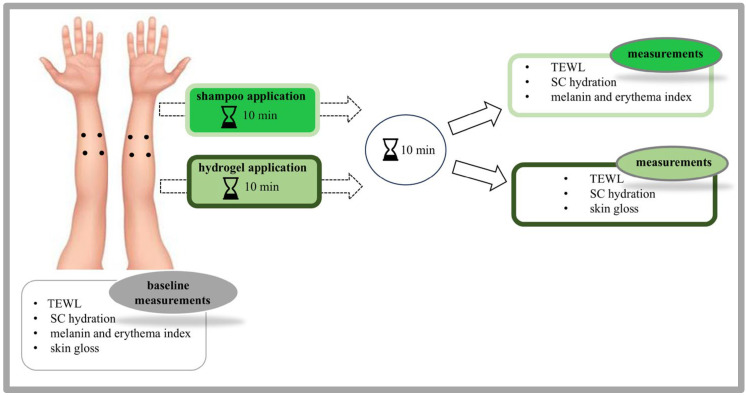
A schematic depiction of the in vivo study process.

Within the study, we assessed the effects of a shampoo as well as a hydrogel with and without whey incorporated on 10 female volunteers. As schematically presents in Figure 7, impact of shampoo was evaluated by measuring TEWL, SC hydration, and erythema and melanin index. In the case of the hydrogel, measurements of TEWL, SC hydration, and skin gloss were conducted. Before the measurements, volunteers were requested to spend at least 30 min in the environmentally controlled room (21.5 ± 1.5 °C, 40.0 ± 5.0% relative humidity) to acclimatise. Throughout acclimatisation and measurements, only one tested individual was present in the room. All subjects had one test area marked on each volar forearm, each with a test area size of 16 cm^2^. Given that two products were tested, the study procedure was structured in two cycles. Specifically, shampoo was first assessed, followed by a free interval of seven days, after which the assessment of the hydrogel was undertaken. All the experimental assessments were performed before formulation exposure (baseline values), followed by formulation exposure for 10 min. After careful removal of the formulation using a cotton swab, the measurements were conducted following an additional 10-min period to ensure that the skin was completely dry and free from any residue of tested product. Statistical analysis of results was carried out using the independent samples Student’s *t*-test with significance tested at the 0.05 level of probability.

##### Transepidermal Water Loss

The entire study was initiated with TEWL measurements, as TEWL is a highly sensitive skin parameter. TEWL was assessed by Tewameter^®^ TM 300 after heating the probe to 32 °C. In each test area, one measurement was performed per assessment time point. Individual measurement was performed for 60 s, with one reading collected per second. The average of ten consecutive readings with the lowest SD represented the TEWL value used for further analysis. The results were expressed in g/hm^2^.

##### Stratum Corneum Hydration

SC hydration measurements were performed by Corneometer^®^ CM 825 (Courage + Khazaka electronic GmbH) in accordance with the protocol assessment time schedule. For each assessment time point, three measurements, which were averaged to obtain the final value, were performed per test area. The results are expressed in arbitrary units (a.u.).

##### Erythema and Melanin Index

Erythema level and melanin content were determined by Mexameter^®^ MX 18 (Courage + Khazaka electronic GmbH) at predetermined time points following study protocol with both values gained within a single measurement in the allocated test areas. For erythema measurement, skin reflection was measured at the wavelengths of 568 nm and 660 nm, corresponding to haemoglobin absorption peaks. For melanin evaluation, the wavelengths of 660 nm and 880 nm are considered, which correspond to different absorption rates of the pigments. The results are expressed in a.u.

##### Skin Gloss

To indirectly assess the cleansing ability of the hydrogel, Skin-Glossymeter GL 200 (Courage + Khazaka electronic GmbH) measurements were performed at time points, as given within study protocol. The gloss value, expressed in a.u., represents the portion of directly reflected light measured with the probe channel, while diffuse channel detects the scattered light [35]. In order to mitigate the impact of an individual’s skin colour on the gloss value, the results are reported considering the diffuse scattering correction (DSC), which is automatically integrated into the measurement system.

## Figures and Tables

**Table 1:** Composition of shampoo formulations.

Component	% (m/m)									
	F1	F2	F3	F4	F5	F6	F7	F8	F9	F10
purified water	88.5	83.5	78.5	73.5	78.5	78.5	78.5	68.5	58.5	48.5
sodium laureth sulfate	5	10	15	20	15	15	15	15	15	15
sodium chloride	2.5	2.5	2.5	2.5	2.5	2.5	2.5	2.5	2.5	2.5
cocamidopropyl betaine	1.5	1.5	1.5	1.5	0.5	2.5	3.0	2.5	2.5	2.5
cocamide diethanolamine	1.5	1.5	1.5	1.5	2.5	0.5	-	0.5	0.5	0.5
propylene glycol	1	1	1	1	1	1	1	1	1	1
whey	/	/	/	/	/	/	/	10	20	30

**Table 2:** Composition of hydrogel formulations.

Component	% (m/m)							
	F1	F2	F3	F4	F5	F6	F7	F8
purified water	80	79.5	79	74	69	69	59	49
decyl glucoside	10	10	10	15	20	10	10	10
glycerol	7.5	7.5	7.5	7.5	7.5	7.5	7.5	7.5
almond oil	2	2	2	2	2	2	2	2
xanthan gum	0.5	1	1.5	1.5	1.5	1.5	1.5	1.5
whey	/	/	/	/	/	10	20	30

**Table 3:** Absolute transepidermal water loss (TEWL) values and changes from baseline after a 10 min treatment with tested formulations.

Formulation	Baseline	95% Confidence Interval	Absolute Value After 10 min	95% Confidence Interval	*p*-Value *	*p*-Value **
shampoo without whey	12.31 ± 3.54	(9.78, 14.84)	13.97 ± 2.85	(11.93, 16.00)	0.05	0.25
shampoo with whey	12.85 ± 2.28	(11.21, 14.48)	13.80 ± 3.88	(11.02, 16.58)	0.22	
hydrogel without whey	12.27 ± 2.89	(10.07, 14.47)	13.58 ± 2.59	(11.72, 15.44)	0.07	0.19
hydrogel with whey	13.62 ± 3.33	(11.24, 16.01)	13.93 ± 1.39	(12.94, 14.92)	0.37	

All data are given in g/hm^2^. N = 10. Data are shown as mean ± SD. * Change from baseline after 10 min, paired *t*-test. ** Difference in baseline vs. after 10 min treatment amongst shampoo/hydrogel with or without whey incorporated.

**Table 4:** Skin capacitance values and changes from baseline after a 10 min treatment with tested formulations.

	Baseline	Absolute Value After 10 min	*p*-Value *	*p*-Value **
shampoo without whey	23.91 ± 6.47	27.18 ± 5.53	0.003	0.29
shampoo with whey	21.87 ± 3.54	25.69 ± 3.63	0.0005	
hydrogel without whey	25.81 ± 5.64	46.75 ± 8.04	5.24 × 10^−7^	0.42
hydrogel with whey	21.97 ± 5.66	43.59 ± 9.37	9.74 × 10^−7^	

All data are given in Corneometer^®^ (Courage + Khazaka electronic GmbH, Köln, Germany) a.u. N = 10. Data are shown as mean ± SD. * Change from baseline vs. after 10 min, paired *t*-test. ** Difference in baseline vs. after 10 min treatment amongst shampoo/hydrogel with or without whey incorporated.

## Data Availability

The original contributions presented in this study are included in the article. Further inquiries can be directed to the corresponding author.
